# Dental plaque-inspired peptide engineered to control plaque accumulation

**DOI:** 10.1016/j.mtbio.2025.101570

**Published:** 2025-02-08

**Authors:** Huixue Wu, Yiran Qin, Kexin Li, Xinning Dai, Minghong Zhou, Zongheng Cen, Yan Li, Zhike Huang, Shuyi Wu

**Affiliations:** a Hospital of Stomatology, Guanghua School of Stomatology, Guangdong Provincial Key Laboratory of Stomatology, Sun Yat-sen University, Guangzhou, 510055, PR China; b Medical Research Institute, Guangdong Provincial People's Hospital (Guangdong Academy of Medical Sciences), Southern Medical University, Guangzhou, 510080, PR China; c Key Laboratory for Polymeric Composite and Functional Materials of Ministry of Education, School of Chemistry, Sun Yat-sen University, Guangzhou, 510275, PR China

**Keywords:** Salivary-acquired peptide, Zwitterionic peptide, Anti-fouling, Plaque accumulation, Tooth surface

## Abstract

Effective control of plaque accumulation is an important strategy for reducing the risk of both localized oral health issues and systemic diseases associated with plaque. However, existing approaches for preventing plaque accumulation exhibit some limitations, such as insufficient compatibility with the oral microbiota and tissues, as well as inconvenience in their use. Herein, inspired by dental plaque, a new class of peptides featuring excellent anti-fouling performance is successfully developed. Our peptides consist of a salivary-acquired peptide with tooth surface-selective adhesion, a zwitterionic peptide with anti-adhesion property, and four proline residues that provide structural rigidity. We conduct a series of progressive experiments, including molecular dynamics simulation and assessments of the anti-fouling performance of our peptides on hydroxyapatite slices, human tooth enamel slices, and ex vivo human teeth. The results demonstrate that our peptides possess the abilities of rapid anchoring on tooth surfaces and effective inhibiting protein and bacterial adhesion. These characteristics enable our peptide to efficiently control plaque accumulation through rinsing or spraying while preserving the balance of the oral microbiota. These findings open an appealing avenue for the development of anti-fouling agents for controlling plaque accumulation on tooth surfaces.

## Graphical abstract

**Figure:**
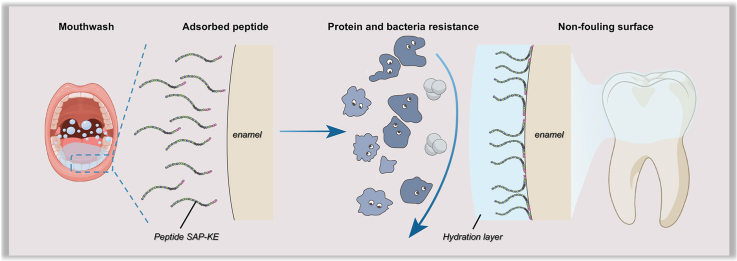

## 1 Introduction

**Fig. 1:**
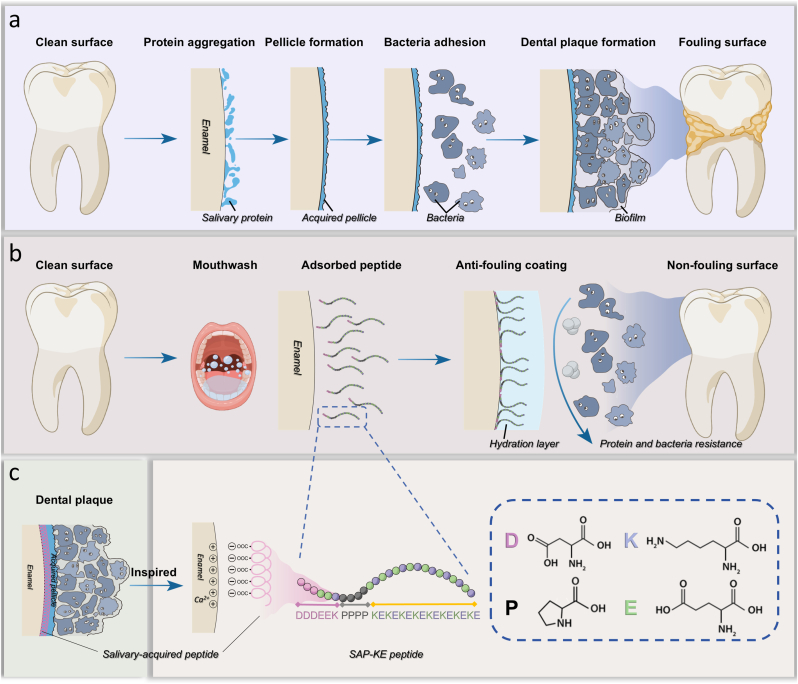
**Schematic illustration of the structure and anti-fouling behavior of dental plaque-inspired SAP-KE coating engineered to effectively prevent plaque accumulation.** (**a**) Even after tooth surfaces are thoroughly cleaned, salivary proteins rapidly adsorb onto the surfaces in the oral cavity, forming an acquired pellicle that subsequently attracts bacteria and ultimately facilitating the formation of dental plaque. (**b**) After cleaning the tooth surfaces, rinsing with SAP-KE solution allows peptides to rapidly adsorb onto the surfaces, forming a protective coating. This coating effectively prevents bacterial and protein adhesion by establishing a robust hydration layer, ultimately inhibiting plaque formation and efficiently maintaining the cleanliness of the tooth surfaces. (**c**) As a biomimetic prototype, natural dental plaque selectively binds to HA of tooth surfaces through the functional component SAP present in acquired pellicle. Inspired by this mechanism, we utilized SAP as the tooth surface-anchoring sequence and the zwitterionic peptide KE sequence as the anti-adhesion sequence. The two sequences are connected by four proline residues, resulting in the successful construction of SAP-KE peptide. This peptide exhibits both rapid binding to HA and anti-fouling properties, offering an innovative approach to dental plaque treatment by mimicking the natural process of plaque formation.

Oral diseases are the most prevalent non-communicable diseases [1]. According to the Global Oral Health Status Report 2022, they affect nearly half of the global population (45 % or 3.5 billion people) throughout the lifespan, from early childhood to old age. The global public and private expenditures on oral health care have reached almost US $390 billion, imposing huge economic burden on many countries [2]. Most infectious oral diseases are closely associated with the accumulation of dental plaque on tooth surfaces (Fig. 1a) [3,4]. The consequences of plaque accumulation are multifaceted, contributing not only to localized oral health issues such as periodontitis [5], caries [6], and even endodontic diseases [7], but also to the exacerbation of various systemic conditions including those affecting the digestive [8,9], respiratory [10,11], nervous [12,13], and cardiovascular systems [14,15]. Of particular concern are vulnerable patients who encounter significant challenges in maintaining regular mechanical tooth brushing due to various factors such as postoperative pain in oral and maxillofacial regions [[16], [17], [18]], limited mouth opening following head and neck radiotherapy [19,20], and reduced ability to perform self-care [21,22]. Additionally, both scientific researchers and underserved populations in resource-limited environments also warrant attention. These groups are at an even greater risk of oral hygiene-related complications. Therefore, how to develop simple yet effective strategies to prevent plaque accumulation on tooth surfaces is not only a critical clinical issue but also an important scientific problem that requires urgent attention.

At present, plaque control and postoperative oral care in clinical practice are primarily achieved by using mouthwash. Chlorhexidine-based mouthwash is recognized as the gold standard for plaque control due to its broad-spectrum antibacterial property [23,24]. However, its frequent use is not recommended, as it may disrupt the oral microbiota [25], alter taste, cause mucosal irritation, and lead to tooth discoloration [26]. Efforts have been made to develop targeted antibacterial agents, such as antibacterial peptides [27] and quorum sensing inhibitors [28,29], to selectively eliminate specific pathogenic bacteria. But the frequent use of these agents, particularly for preventive purposes, still poses a potential risk of disrupting the oral microbiota. To address these challenges, hydrophilic polymers are introduced onto the tooth surface to create anti-adhesion coatings [30,31]. However, this approach requires prolonged contact with the tooth surface or irradiation with specific wavelengths of light to ensure stable bonding to the enamel. Its application highly depends on both clinic operations and patient's ability to maintain adequate mouth opening. In addition, a sprayable superhydrophobic anti-fouling agent based on organic adhesive and inorganic composite has been proposed [32]. Yet, the introduction of inorganic fillers may cause issues such as occlusal interference and accelerated tooth wear. Therefore, there is currently no ideal tooth surface anti-fouling agent available. Agents that effectively combine oral environmental compatibility, excellent anti-fouling performance, rapid and firm adhesion to the tooth surface, and convenient usability are expected to fulfil the diverse needs of oral health care with high development value and broad application prospects.

Herein, inspired by dental plaque, we have successfully developed a class of rinseable or sprayable tooth surface anti-fouling agent that can perfectly meet the above requirements (Fig. 1b). As a prototype of biomimetic design, the formation of natural dental plaque originates from the rapid and firm binding of salivary-acquired peptide (SAP) from acquired pellicle to hydroxyapatite (HA) on the tooth surface, followed by the continuous recruitment of oral microbiota [[33], [34], [35], [36], [37]]. Inspired by this natural process, we utilize DDDEEK sequence of SAP as the tooth surface anchoring sequence [[38], [39], [40], [41]], employ a zwitterionic peptide (composed of alternating lysine (K) and glutamic acid (E) residues) with stable hydration property as the anti-adhesion sequence, and link these two sequences with four proline (P) residues, resulting in SAP-KE tooth surface anti-fouling agent (Fig. 1c). In this design, SAP stabilizes its binding with the calcium ions of HA after 50 ns of contact through electrostatic interactions, exerting a strong and selective adhesion effect on the tooth surface. KE sequence, composed of alternating positively and negatively charged amino acids, forms a stable hydration layer to effectively hinder protein and bacterial adhesion [[42], [43], [44], [45], [46], [47]]. The intervening proline residues provide structural rigidity and enhance the extensibility and monolayer packing density of the peptide chains, contributing to the effectiveness of the agent [48]. Based on this well-orchestrated design, our SAP-KE significantly reduces plaque coverage on ex vivo human teeth after a single application, maintaining a lower plaque area after 7 days compared to the bare tooth surface after 1 day. When the application frequency of SAP-KE is increased to once every two days, the plaque coverage on the tooth surface is reduced by 70 % after 7 days, compared to the bare surface. In brief, our dental plaque-inspired SAP-KE can rapidly form a stable anti-fouling coating on the tooth surface, thereby efficiently preventing plaque accumulation. Our strategy would not only provide new ideas for efficiently maintaining oral hygiene, but also offer a convenient approach to reduce the risk of oral problems and systemic diseases associated with plaque accumulation. Furthermore, it would also serve as a typical demonstration for applying the strategy of ‘a taste of your own medicine’ in the field of biomedicine and even scientific research.

## 2 Materials and methods

### 2.1 Synthesis of peptides and characterizations of peptide coated HA

#### 2.1.1 Preparations of peptides and physicochemical characterizations of peptide coated HA

All the peptides were prepared via a standard Fmoc-based solid-phase peptide synthesis strategy by Genscript (China). The purities and the mass-to-charge ratios of KE, SAP and SAP-KE peptides were analyzed by high performance liquid chromatography (HPLC; LC2030, Shimadzu, Japan) and mass spectra (LCMS2020, Shimadzu, Japan), respectively. The HA slices (8.4 mm in diameter and 1.8 mm in thickness; Recongene, China) were polished with SiC sandpapers from 800#, 1000#, 1500# to 2000#. The ready-prepared aqueous solutions of KE, SAP, and SAP-KE (5.00 mg/mL) were separately coated on the surfaces of polished HA slices for 10 min, rinsed with deionized water for three times, and air-dried. Some of SAP-KE coated HA slices were immersed in artificial saliva (AS; pH = 7.0; Solarbio, China) for 7 days or modified AS (pH = 5.5). The HA slices were measured by an attenuated total reflection Fourier transform infrared (ATR-FTIR) spectrometer (TENSOR27, Bruker, Germany) to analyze the chemical groups of the samples. Water contact angles were measured using a drop shape analyzer (DSA-XROLL, Betop Scientific, China) to test the hydrophilicity of the surfaces. The charge distribution and the relative potential of the coatings were measured by a Kelvin probe force microscope (KPFM; Dimension Fastscan, Bruker, Germany). Scanning electron microscopy (SEM; EVO MA10, Carl Zeiss, Germany) was used to observe the surface morphology of the coating. Transmission electron microscopy (TEM; HT7800, Hitachi, Japan) was used to observe the size of SAP-KE peptide. Roughness measurements were performed by atomic force microscopy (AFM; Dimension Fastscan, Bruker, Germany).

#### 2.1.2 Adsorption of SAP-KE on HA powder

SAP-KE aqueous solutions with different concentrations ranging from 0.50 to 3.00 mg/mL were prepared. The corresponding absorbances were measured using a UV spectrophotometer (Nanodrop 2000, Thermo Fisher Scientific, USA) at a wavelength of 220 nm and a standard curve was constructed. 50.00 mg HA powder was added into 1 mL (denoted as *V*) SAP-KE aqueous solution with different concentrations (0.50–3.00 mg/mL; denoted as *C*_0_). Each mixture was stirred at room temperature for 24 h and centrifuged at 10000 rpm for 4 min. The supernate was collected using a 0.22 μm filter and the absorbance was measured using a UV spectrophotometer at a wavelength of 220 nm. The concentrations of SAP-KE aqueous solutions after the adsorption process (denoted as *C*_1_) were obtained according to the standard curve. The adsorption amount and rate of SAP-KE on HA powder were calculated according to Equations (1), (2):

(1) $$\text{Adsorption}\;\text{amount}=V\left(C_{0}-C_{1}\right)$$

(2) $$\text{Adsorption}\;\text{rate}\;\left(\%\right)=\frac{V\left(C_{0}-C_{1}\right)}{VC_{0}}\times 100\%$$

#### 2.1.3 Biocompatibility evaluations

The cytotoxicity of SAP-KE was evaluated in MC 3T3 cells (iCell Bioscience Inc, China) using the cell counting kit‐8 (CCK-8; Dojindo, Japan). Cells were seeded in a 96-well plate at a density of 5000 cells per well in 100 μL α-MEM medium. After 24 h of incubation, the medium was replaced with 100 μL fresh medium containing 2.00 mg/mL SAP-KE. Medium without SAP-KE and without cells served as the control group and blank group, respectively. The optical density (OD) of the incubation solution was measured at 450 nm using a microplate reader (Epoch 2, BioTek, USA) on days 1, 2, and 3. The cell viability with SAP-KE was calculated according to Equation (3):

(3) $$\text{Cell}\;\text{viability}\;\left(\%\right)=\frac{{\text{OD}}_{\text{SAP}‐\text{KE}\;}-{\text{OD}}_{\text{blank}}}{{\text{OD}}_{\text{control}}-{\text{OD}}_{\text{blank}}}\times 100\%$$

To measure the effect of SAP-KE on the proliferation activities of human oral flora, dental plaques were collected from the tooth surfaces of volunteers and transferred to brain heart infusion (BHI) culture medium. The procedures were performed under the permission of the Medical Ethics Committee (MEC) of Hospital of Stomatology, Sun Yat-sen University (No. KQEC-2024-105-01). According to the manufacturer's instructions, *Streptococcus mutans* (*S. mutans*; ATCC 700610, Guangdong Microbial Culture Collection Centre, China) with known different ratios of live bacteria were stained with LIVE/DEAD *Bac*Light Bacterial Viability Kit (Thermo Fisher Scientific, USA) and the corresponding green to red fluorescence ratio values were measured using a fluorescence microplate reader (Biotech-Synergy-H1, BioTek, USA) to obtain a standard curve. Bacteria in the plaque were cultured in medium containing 2.00 mg/mL SAP-KE and medium without SAP-KE served as the control group. On days 1, 2, and 3, the bacteria were stained with LIVE/DEAD *Bac*Light Bacterial Viability Kit and the fluorescence intensity was detected at wavelengths of 530 nm and 630 nm with the excitation wavelength centered at 485 nm. The values of green and red fluorescences were recorded and the ratios of live bacteria representing bacteria viability in the plaque were obtained according to the standard curve.

### 2.2 Molecular dynamics simulation to evaluate the anchoring and hydration abilities of SAP-KE peptide

The three-dimensional structure of SAP-KE peptide was predicted using AlphaFold Protein Structure Database and constructed by PyMOL 2.4 (DeLano Scientific, USA) software. HA (001) facet was used in modeling HA surface. The Charmm 36 all-atom force field was used in conjunction with the Interface Force Field for HA molecules and the TIP3P water model for water molecules. Molecular dynamics simulation was conducted under constant temperature and pressure with periodic boundary conditions using the Gromacs 2019.6 program. The steepest descent method was used to minimize the energy of the system to eliminate close atomic contacts. Molecular dynamics simulation was then performed in both canonical and isobaric-isothermal ensembles at 298.15 K and 1 bar. A 100 ns simulation of the system was conducted and the conformations were saved every 10 ps. The results were analyzed using the analysis program in Gromacs 2019.6 package and Visual Molecular Dynamics.

### 2.3 Evaluation of the anti-fouling performance against protein on HA

For visual analysis, bare, KE, SAP, and SAP-KE coated HA slices were immersed in fluorescein isothiocyanate-labeled bovine serum albumin (FITC-BSA; Solarbio, China) solution (0.10 mg/mL), incubated at 37 °C for 90 min, washed with phosphate-buffered saline (PBS) for 3 times, and dried at room temperature. The attached FITC-BSA was observed under a confocal laser scanning microscope (CLSM; FV3000, Olympus, Japan).

For quantitative analysis, bare, KE, SAP, and SAP-KE coated HA slices were immersed into BSA (Amresco, USA) solution (5.00 mg/mL) and incubated at 37 °C for 2 h with continuous shaking at 200 rpm. The unbound proteins were removed by rinsing with deionized water and the bound proteins were detached from the surfaces by ultrasonication for 15 min in 1 mL deionized water. The mount of separated BSA of bare HA group (*m*_HA_) and peptide coated HA groups (*m*_treatment_) was measured by BCA™ protein assay kit (Jiangsu Cowin Biotech Co., Ltd., China). The protein anti-fouling rates were calculated according to Equation (4):

(4) $$\text{Protein}\;\text{anti}‐\text{fouling}\;\text{rate}\;\left(\%\right)=\frac{m_{\text{HA}}-m_{\text{treatment}}}{m_{\text{HA}}}\times 100\%$$

### 2.4 Evaluation of the anti-fouling performance against bacteria on HA

The anti-fouling performance against bacteria of various coatings on HA was evaluated using *S. mutans* through SEM and AFM. 400 μL of *S. mutans* suspension (1 × 10^6^ CFU/mL) was added onto the surfaces of bare, KE, SAP, and SAP-KE coated HA slices and cultured in BHI medium at 37 °C for 24 h. The slices were then rinsed with PBS, immersed in 2.5 % glutaraldehyde overnight at 4 °C, and dehydrated in an ascending ethanol gradient. After air-drying, the samples were coated with gold and observed by SEM.

To detect the adhesion force of bacteria on the surface of SAP-KE coating, a glass bead was used as an intermediate to connect the bacteria to the tip of the NP-O10 probe (Bruker, Germany). Glass beads sized 5–10 μm were incubated into 0.01 % poly-L-lysine (Shanghai Yuanye Bio-Technology Co., Ltd., China) at room temperature for 8 h and centrifuged to obtain a precipitate. The modified beads were resuspended with 1000 μL *S. mutans* bacterial suspension (1 × 10^8^ CFU/mL), kept in the incubator for 15 min, and centrifuged at 2000 rpm for 5 min to obtain a precipitate. To confirm the adhesion of live bacteria to the surfaces of the beads, *S. mutans* adhered to the beads were stained using LIVE/DEAD *Bac*Light Bacterial Viability Kit and subsequently observed under CLSM (LSM 980, Carl Zeiss, Germany). The tip of probe was coated with epoxy resin and immersed in the suspension of *S. mutans* modified beads for 2 min to obtain a bacteria-modified probe, which was immediately used for force measurement. Force measurements were performed using AFM in contact mode. The scanning area was 5 μm × 5 μm and the scanning rate was set to 1 Hz. The surfaces of the samples (bare and SAP-KE coated HA slices incubated in AS for 0, 3, and 7 days) were covered with PBS to ensure that the entire test procedure was conducted in a liquid environment. The bacteria-modified probe was approached to the surface of the sample and then retracted. The adhesion force between the bacteria and the surface caused the cantilever to bend toward the sample, requiring the probe to move a certain distance before separation from the surface. During this process, the force-distance curve and the maximum adhesion force were recorded.

### 2.5 Assessment of the anti-fouling performance against biofilm on tooth enamel

The anti-fouling performance against biofilm of various coatings on tooth enamel was evaluated using *S. mutans* through fluorescence staining. Freshly extracted intact human premolars and molars were collected and stored in a 1 % thymol solution. Meanwhile, unstimulated whole saliva was collected from 5 healthy adult volunteers, mixed thoroughly, and centrifuged at 12000 rpm for 20 min at 4 °C. The supernatant was then filtered and stored at 4 °C for subsequent use. These procedures were performed under the permission of the MEC of Hospital of Stomatology, Sun Yat-sen University (No. KQEC-2024-109-01). The crowns were sectioned longitudinally to obtain enamel slices. The enamel surfaces of the slices, set as the working side, were ground flat and polished with SiC sandpaper from 800#, 1000#, 1500# to 2000#. The enamel slices were immersed in peptide (KE, SAP, and SAP-KE) solutions for 10 min, followed by washing with deionized water to complete the surface modification. Both the modified and bare enamel slices were then immersed in saliva and co-cultured with *S. mutans* (400 μL, 1 × 10^6^ CFU/mL) in BHI medium containing 1 % (w/v) sucrose for 24 h. The bacteria were stained by a LIVE/DEAD *Bac*Light Bacterial Viability Kit and the biofilm on the enamel surface was observed using CLSM. Quantitative analyses of biofilm thickness and biomass were performed using Comstat v.2.1 software.

### 2.6 Measurement of the anti-fouling performance against dental plaque on ex vivo human teeth

The anti-fouling performance against dental plaque on ex vivo human teeth was assessed using complex bacterial flora through plaque indicator staining. After signing informed consents, freshly extracted intact human premolars were collected and stored in a 1 % thymol solution. Dental plaques were collected from the tooth surfaces of two volunteers and immediately transferred to the culture medium. These procedures were conducted under the permission of the MEC of Hospital of Stomatology, Sun Yat-sen University (No. KQEC-2024-109-01 and No. KQEC-2024-105-01). The ex vivo teeth were immersed in peptide (KE, SAP, and SAP-KE) solutions for 10 min and washed with deionized water to finish the surface modification. The modified and bare teeth were placed in a 24-well culture plate and co-cultured with plaque suspension in BHI culture medium under alternating dynamic and static cycles for 1, 3, and 7 days. In dynamic condition, stirring was conducted at 150 rpm for 12 h at 37 °C, while in static condition, stirring was ceased and the culture was maintained at 37 °C for 12 h. The surfaces of the crowns were stained using a plaque indicator (Ci Medical, Japan), rinsed with PBS, air-dried, and recorded by digital photos. To investigate the anti-fouling effect after increasing coating frequency, SAP-KE coated teeth were co-cultured with plaque suspension and the coating was replenished for 10 min on days 2, 4, and 6. Plaque staining was performed on day 7 using a plaque indicator, followed by taking digital photos for documentation. The total enamel area of each tooth in the captured photo was selected using Adobe Photoshop 2024 (Adobe Systems, USA). Green colored channel was selected to identify the stained area. All stained areas were highlighted by ‘Select Similar’ and then saved. The numbers of pixels corresponding to the total enamel area and the stained area were measured in each photo. The percentage plaque index (PPI) was calculated according to Equation (5):

(5) $$\text{PPI}\;\left(\%\right)=\frac{\text{total}\;\text{pixels}\;\text{of}\;\text{stained}\;\text{area}}{\text{total}\;\text{pixels}\;\text{of}\;\text{enamel}\;\text{area}}\times 100\%$$

### 2.7 Statistical analysis

All experiments were repeated at least three times. Statistical analysis was performed by GraphPad Prism 8 software and data were expressed as mean ± standard deviation (SD). Data with homogeneity of variance were analyzed by Student's *t*-test or one-way analysis of variance (ANOVA) followed by least significant difference (LSD) multiple comparisons. Data with heterogeneity of variance were analyzed by Kruskal-Wallis's nonparametric test. The significance level was set as *P* < 0.05.

## 3 Results and discussion

### 3.1 Synthesis of peptides and characterizations of peptide coated HA

KE, SAP and SAP-KE are prepared by a standard Fmoc-based solid-phase peptide synthesis strategy. SAP-KE is designed as a linear structure in which amino acids are directly linked by peptide bonds, giving it both high activity and stability. High-performance liquid chromatography analysis shows that the purities of KE, SAP and SAP-KE are 98 %, 97 %, and 97 %, respectively (Fig. S1). The mass spectrometry analyses (Fig. S2)reveal that the values are in good agreement with the theoretical molecular weights of KE (2076.32 Da), SAP (749.68 Da) and SAP-KE (3196.44 Da). These results confirm the successful synthesis of peptides.

**Fig. 2:**
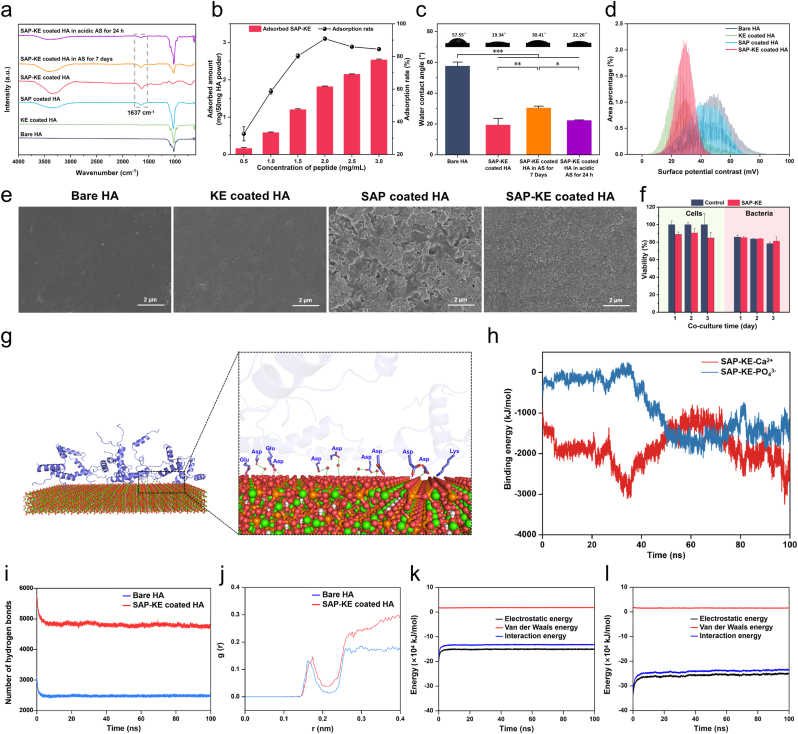
**Characterizations of peptide coatings and molecular dynamics simulation of the adhesion and hydration capabilities of SAP-KE peptide on HA surface**. (**a**) ATR-FTIR spectra of bare HA, KE coated HA, SAP coated HA, SAP-KE coated HA, SAP-KE coated HA in AS for 7 days, and SAP-KE coated HA in modified AS (pH = 5.5) for 24 h. (**b**) Adsorption amount and rate curve of SAP-KE on HA powder (n = 4 independent samples). (**c**) Water contact angles of bare HA, SAP-KE coated HA, SAP-KE coated HA in AS for 7 days, and SAP-KE coated HA in modified AS (pH = 5.5) for 24 h (n = 3 independent samples; ANOVA followed by LSD multiple comparisons; ∗∗*P* < 0.01, ∗∗∗*P* < 0.001). (**d**) Frequency histograms of surface potentials of bare, KE, SAP and SAP-KE coated HA. (**e**) SEM images of bare, KE, SAP and SAP-KE coated HA. (**f**) The viabilities of MC 3T3 cells and plaque bacteria cultured in the medium without or with 2 mg/mL SAP-KE after 1, 2, and 3 days (n = 3 independent samples). (**g**) Snapshot of SAP-KE molecules on HA substrate (001) at the end of molecular dynamics simulation.(O = red, Ca = green, P = orange, N = blue) (**h**) The binding energies of SAP-KE with Ca^2+^ and PO_4_^3−^ over 100 ns simulation time. (**i**) The number of hydrogen bonds between water molecules and SAP-KE coated HA or bare HA. (**j**) RDFs of water molecules around SAP-KE coated HA and bare HA. (**k, l**) The interaction energies between water molecules and bare HA (**k**) or SAP-KE coated HA (**l**). All error bars = SD; data are presented as mean values ± SD. (For interpretation of the references to color in this figure legend, the reader is referred to the Web version of this article.)

Considering that human enamel contains up to 96 % HA [49], HA slices are employed to simulate the tooth surface and serve as the substrate for peptide coatings. HA slices are separately immersed in KE, SAP and SAP-KE aqueous solutions for 10 min to obtain the samples of KE, SAP and SAP-KE coated HA. These samples, as well as bare HA and SAP-KE coated HA in AS for 7 days, are observed by ATR-FTIR spectrometer. As illustrated in Fig. 2a, amide vibration peaks around 1637 cm^−1^ [50], attributed to the carbonyl groups of peptides, are observed in the SAP and SAP-KE coated samples. In contrast, these peaks are absent in the HA and KE coated samples, indicating that SAP plays a critical role in anchoring KE to HA surface. After soaking in AS (pH = 7.0) for 7 days or AS (pH = 5.5) for 24 h, the characteristic peak of SAP-KE remains detectable on the surfaces of the samples, demonstrating its persistent adsorption on HA and highlighting its potential for application in normal and acidic oral environments. To evaluate the optimal concentration of SAP-KE, we mix SAP-KE aqueous solutions at varying concentrations with 50.00 mg HA powder and calculate both the adsorption amount and the adsorption rate of SAP-KE. Fig. 2b shows that the adsorption amount of SAP-KE on HA powder gradually raises as the peptide concentration increases, conforming well to the Langmuirmodel (*R*^*2*^ > 0.95). This indicates that the adsorption process of SAP-KE on HA follows a monolayer adsorption mechanism [51]. The peptide coating on HA is likely a single molecular layer, with the coating thickness approximately equal to the size of SAP-KE peptide. Through molecular simulation, we obtain that the theoretical size of SAP-KE peptide is around 4.20 nm (Fig. S3a). To confirm this, we observe the peptide using TEM, and the observed particle size is consistent with the simulation result (Fig. S3b), supporting our hypothesis. When the peptide concentration is 2.00 mg/mL, the adsorption rate of SAP-KE on HA reaches its peak (91 %). Therefore, 2.00 mg/mL is selected as the working concentration for further experiments. Water contact angle measurements are performed to detect the wettability of various surfaces (Fig. 2c). The average water contact angle of SAP-KE coated HA (19.34°) is significantly lower than that of bare HA (57.55°), indicating that SAP-KE can effectively enhance the hydrophilicity of the substrate surface. After immersing the samples in AS (pH = 7.0) for 7 days and AS (pH = 5.5) for 24 h, the hydrophilicity of the SAP-KE coated HA surfaces still remains at a high level, providing a good foundation for the coating to maintain its anti-fouling ability in normal and acidic oral environments. The surface potential mapping images reflecting the relative potential differences between various substrate positions and the probe are obtained by KPFM [52] (Fig. S4). As shown in Fig. 2d, the average potential contrasts of SAP-KE (28.3 mV) and SAP (40.0 mV) coated HA are significantly lower than that of bare HA slice (52.1 mV). This indicates that the surfaces of SAP-KE and SAP coatings carry negative charges comparing to bare HA surface, which is beneficial for repelling negatively charged bacteria.

To investigate the surface morphologies of the coatings, we employ SEM to examine the surface of each sample. As shown in Fig. 2e, SAP-KE peptides are more uniformly distributed on the HA slice to form a homogeneous coating compared to SAP coating. In sharp contrast, KE peptide has little affinity for HA due to the lack of specific adhesion sequence and its coating shows a similar appearance to that of bare HA surface. We further evaluate the cell compatibility of SAP-KE peptide using CCK-8 method, with deionized water as control. The results indicate that the cell proliferation trends in both SAP-KE and control groups are similar on days 1, 2, and 3 (Fig. 2f), demonstrating the favorable cell compatibility of SAP-KE. To investigate the effect of SAP-KE peptide on normal oral flora, we co-culture complex bacteria from the dental plaque of volunteers with SAP-KE aqueous solution (using deionized water as a control) for 3 days and measure the proportions of dead bacteria. The results indicate no significant difference between SAP-KE group and the control group (Fig. 2f), suggesting that SAP-KE does not significantly promote bacterial proliferation or kill bacteria. Therefore, SAP-KE has good cell compatibility and does not disrupt the balance of normal oral microbiota.

### 3.2 Molecular dynamics simulation to evaluate the anchoring and hydration abilities of SAP-KE peptide

Although the aforementioned results clearly demonstrate that SAP-KE peptide can stably anchor to the surface of HA slice, its anchoring process requires further exploration from a microscopic perspective. Therefore, we employ molecular dynamics simulation to analyze the interactions between SAP-KE peptide and HA surface. During the simulation, we observe that SAP-KE peptides, initially randomly distributed above HA, rapidly aggregate on HA surface, forming a stable composite system by approximately 50 ns (Fig. S6). Most of these peptides bind with HA surface at the N-terminus (starting from SAP) through O or N atoms of negatively charged aspartic acid and glutamic acid, allowing the peptides to anchor stably to HA surface (Fig. 2g). To further investigate the binding mechanism, the binding energy of the electrostatic interaction between SAP-KE and Ca^2+^, as well as that of hydrogen bonding between SAP-KE and PO₄^3−^, is analyzed. The results show that the former is −2014.97 kJ/mol and the latter is −303.31 kJ/mol during the first 50 ns (Fig. 2h). This indicates that the electrostatic interaction between SAP-KE and Ca^2+^ plays a dominant role in the early adhesion of SAP-KE to the tooth surface. These findings fully demonstrate that SAP-KE peptides can rapidly and firmly adhere to HA through SAP.

The KE sequence, characterized by alternating positively charged lysine (Lys, K) and negatively charged glutamic acid (Glu, E), is one of the most representative zwitterionic peptides [42,43]. This sequence has already been designed for applications in biosensors [44,45], drug delivery carriers [46], and self-cleaning surfaces [47], owing to its outstanding anti-fouling property. The key to the anti-fouling action of zwitterionic peptides lies in their ability to form a robust hydration layer with water molecules through hydrogen bonds. This hydration layer serves as a physical and energetic barrier, effectively preventing the nonspecific adhesion of proteins and bacteria [[53], [54], [55]]. Therefore, we evaluate the hydration ability of SAP-KE coated HA from a microscopic perspective using molecular dynamics simulation. From the perspective of hydrogen bonding interactions, Fig. 2i shows that the number of hydrogen bonds formed between the simulated SAP-KE coated HA and water molecules (4819.46) is significantly higher than that formed between bare HA and water molecules (2484.52). In terms of water molecule distribution, radial distribution function (RDF) [56] results indicate that, within the observation range of 0.4 nm, SAP-KE coated HA exhibits a higher RDF peak (0.16 vs. 0.14) and a greater peak distance (0.17 nm vs. 0.16 nm) compared to bare HA (Fig. 2j). These results demonstrate that SAP-KE coated HA comes into contact with more water molecules compared to bare HA. Regarding interaction energy, the average energy between SAP-KE coated HA and water molecules (−24.18 × 10⁴ kJ/mol) is significantly higher than that between bare HA and water molecules (−13.27 × 10⁴ kJ/mol) (Fig. 2k and l). These indicate that the introduction of SAP-KE peptides enhances the binding affinity between the system and water molecules. The results together demonstrate that the introduction of SAP-KE peptides on HA surface enhances the system's ability to attract more water molecules, thereby forming a robust hydration layer that is beneficial for preventing the adhesion of proteins and bacteria.

### 3.3 Anti-fouling performances of SAP-KE coating against protein and bacteria on HA

**Fig. 3:**
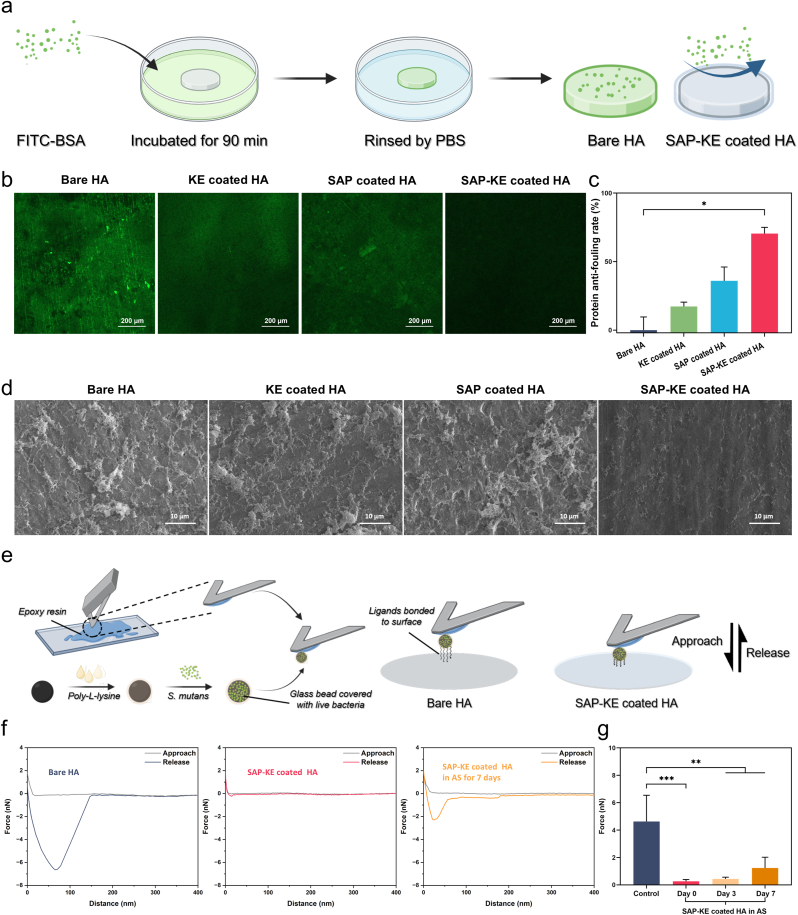
**Anti-fouling performancesof peptide coatings against protein and bacteria on HA**. (**a**) Schematic diagram of using FITC-BSA to test the anti-fouling abilities of peptide coatings. (**b**) Fluorescent images showing the distributions of FITC-BSA on the surfaces of bare, KE, SAP and SAP-KE coated HA (green for FITC-BSA). (**c**) Protein anti-fouling rates of bare, KE, SAP and SAP-KE coated HA (n = 3 independent samples; Kruskal-Wallis's nonparametric test). (**d**) SEM images exhibiting the distributions of *S. mutans* on the surfaces of bare, KE, SAP and SAP-KE coated HA. (**e**) Schematic diagram of preparing bacteria‐modified probe and measuring adhesion force via AFM. (**f**) Force-distance curves of bare HA, SAP-KE coated HA, and SAP-KE coated HA in AS for 7 days. (**g**) The maximum adhesion forces between *S. mutans* and bare HA and SAP-KE coated HA in AS for 0, 3, and 7days (n = 3 independent samples; ANOVA followed by LSD multiple comparisons). All error bars = SD; data are presented as mean values ± SD; ∗*P* < 0.05, ∗∗*P* < 0.01, ∗∗∗*P* < 0.001. (For interpretation of the references to color in this figure legend, the reader is referred to the Web version of this article.)

Bacteria, proteins, and food debris can easily adhere to the surfaces of teeth to form plaque, with protein adhesion serving as the initial step [57,58]. Therefore, preventing the non-specific adsorption of proteins is crucial for inhibiting the aggregation of dental plaque on tooth surface. BSA is used as a representative protein to evaluate the anti-fouling performance of peptide coatings on HA slices. To visually observe protein adhesion, HA slices with various coatings are incubated with FITC-BSA for 90 min, rinsed with PBS, and examined using CLSM (Fig. 3a). As shown in Fig. 3b, SAP-KE coated HA surface exhibits almost no green fluorescence, while bare HA surface shows strong green fluorescence. Both SAP and KE coated HA surfaces also exhibit considerable green fluorescence. We further use BCA protein assay kit to quantitatively analyze the adsorbed BSA on HA. The results show that the protein anti-fouling rate of SAP-KE coated HA group is 70 %, significantly higher than those of the other groups (Fig. 3c). These results indicate that SAP-KE coating can significantly reduce non-specific protein adsorption on HA surface, thereby preventing subsequent protein-dependent bacterial adhesion.

The desired function of our coating is to resist bacterial adhesion. Here, we choose *S. mutans* as the model bacterium, which is one of the most important bacteria in oral biofilm formation [59,60], to evaluate the coating's effectiveness in preventing bacterial attachment. As shown in SEM images, only scattered bacteria are observed on the surface of SAP-KE coated HA, whereas a large number of bacteria aggregate and form thick biofilms on the surfaces of bare, KE, and SAP coated HA (Fig. 3d). We further analyze the remarkable anti-fouling property of SAP-KE coating from the perspective of bacterial adhesion mechanics. *S. mutans*-modified silica bead is attached to the tip of an AFM probe by epoxy resin, and direct adhesive interactions between the bacteria and different coating surfaces are detected in a liquid medium [61](Fig. 3e). As shown in the force-distance curves, the retraction curve of bare HA exhibits a large separation distance (17.06 nm) and a high adhesion force (4.63 nN) between the bacteria and the HA surface. In stark contrast, SAP-KE coated HA surface shows a significant reduction in both separation distance (108.20 nm) and adhesion force (0.26 nN), which is only about one-sixth and one-seventeenth of those of bare HA, respectively. When SAP-KE coated HA is immersed in AS for an extended period, the adhesion strength increases slightly, reaching 1.23 nN after 7 days, but it still remains lower than that of the bare HA surface (Fig. 3f and g). The observed results can be explained as follows. Bacterial adhesion relies on the binding of macromolecules from the bacterial cell wall to a substrate [62]. The hydration layer formed on the SAP-KE coating surface impedes the interaction between the bacterial cell wall and the substrate, providing effective resistance to initial bacterial adhesion and facilitating the detachment of adhered bacteria. This disruption interferes with key steps in biofilm formation. With the continuous action of various factors, such as surrounding oxygen, divalent cations in AS, and proteases produced by bacteria, the peptides gradually degrade [63,64], which, to some extent, compromises the long-term anti-fouling efficacy of the coating. Taken together, SAP-KE coating demonstrates excellent anti-fouling property and reasonable durability against bacteria.

### 3.4 Anti-fouling performance of SAP-KE coating against biofilm on human tooth enamel

**Fig. 4:**
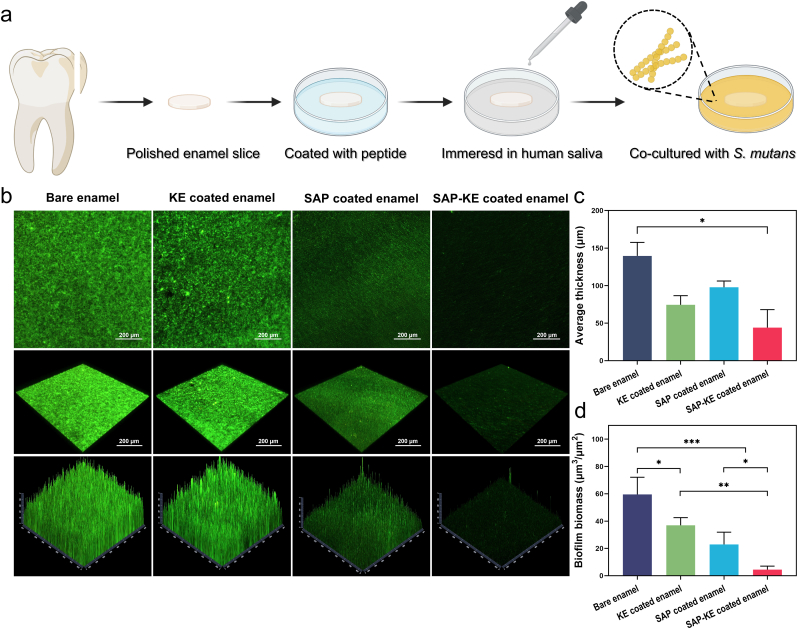
**Anti-fouling performance of peptide coatings against biofilm on human tooth enamel.** (**a**) Schematic diagram of the preparation of peptide coated human enamel slice and the establishment of *S. mutans* biofilm on the slice surface. (**b**) Fluorescent images displaying the formation of *S. mutans* biofilm on the surfaces of bare, KE, SAP and SAP-KE coated enamel slices (green for live bacteria, red for dead bacteria); (**c**, **d**) The average thicknesses (c) and the biomass (d) of the biofilm formed on the surfaces of bare enamel slices and enamel slices with different coatings (n = 3 independent samples; ANOVA followed by LSD multiple comparisons). All error bars = SD; data are presented as mean values ± SD; ∗*P* < 0.05, ∗∗*P* < 0.01, ∗∗∗*P* < 0.001). (For interpretation of the references to color in this figure legend, the reader is referred to the Web version of this article.)

The above favorable results prompt us to analyze the outstanding anti-fouling advantage of our coating on human tooth enamel. Each enamel slice is cut from extracted human teeth, soaked in a peptide solution to form a surface coating, immersed in real human saliva to simulate the oral cavity environment, followed by co-culturing with *S. mutans* for 24 h to allow biofilm formation [65] (Fig. 4a). The fluorescent staining results of live/dead bacteria indicate that KE, SAP, SAP-KE coated enamel slices, similar to bare enamel slices, exhibit no obvious dead bacteria on their surfaces, indicating that the peptide coatings have no notable effect on bacterial activity. As shown in Fig. 4b, thick and dense biofilms can be observed on both bare and KE coated enamel surfaces. Although KE peptide has anti-fouling effect, it is difficult to stay on the enamel surface and exert its effect due to the lack of adhesion sequence. In contrast, SAP-KE coated enamel surface shows a small amount of biofilm, significantly lower than that on SAP coated enamel surface. We further use Comstat software to quantitatively analyze the biomass and thickness of the formed biofilm [66]. The results show that, compared to bare enamel group, the biomass of SAP-KE coated enamel group decreases by 92 %, and the average thickness decreases by 61 % simultaneously (Fig. 4c and d). The above results collectively demonstrate that SAP-KE peptide, composed of SAP anchoring sequence and KE anti- adhesion sequence, can leverage the advantages of both sequences, efficiently providing anti-fouling effects on the enamel surface while remaining compatible with the microbial community.

### 3.5 Anti-fouling performance of SAP-KE coating against dental plaque on ex vivo human teeth

**Fig. 5:**
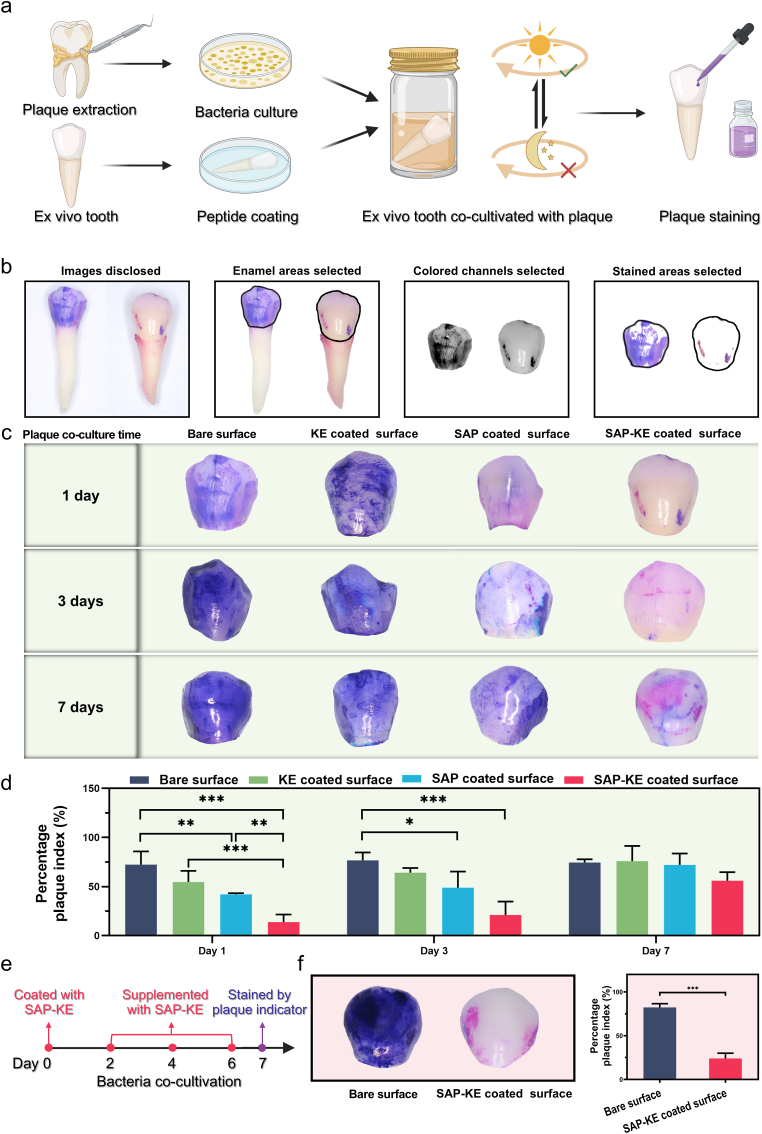
**Anti-fouling performance of peptide coatings against dental plaque on ex vivo human teeth.** (**a**) Schematic diagram of anti-fouling test of peptide coating against complex oral flora on the surface of ex vivo human tooth under the condition of dynamic and static alternating cycles. (**b**) The assessment process of dental plaque coverage on the tooth surface for PPI calculation. (**c,d**) Digital photos of the plaque staining (c) and the corresponding PPI quantitative analyses (d) of ex vivo tooth surfaces without and with single different coatings after co-culturing with the suspension of dental plaque for 1, 3, and 7 days (n = 3 independent samples; ANOVA followed by LSD multiple comparisons). (e) Schematic timeline of experimental design for evaluating the effect of increased SAP-KE coating frequency on tooth surface cleaning maintenance. (f) Digital photos of plaque staining and the corresponding PPI quantitative analyses of bare tooth surface and tooth surface with supplemented SAP-KE coating after co-culturing with dental plaque suspension for 7 days (n = 3 independent samples; Student's *t*-test). All error bars = SD; data are presented as mean values ± SD; ∗*P* < 0.05, ∗∗*P* < 0.01, ∗∗∗*P* < 0.001.

The above experiments demonstrate that SAP-KE coating exhibits excellent anti-fouling properties against proteins, *S. mutans* and its biofilm. To further simulate oral conditions, ex vivo human teeth with or without peptide coatings are co-cultured with human dental plaque (containing over 500 species of bacteria [67]) under alternating dynamic and static cycles to mimic the human oral environment during the alternation of day and night (Fig. 5a). The amount and freshness of the plaque on the tooth surfaces are visually assessed using plaque indicator staining, and corresponding digital photos are recorded. The captured photos are analyzed using Photoshop software to extract the pixels representing the total enamel area and the stained area, from which PPIis calculated [68] (Fig. 5b). The results in Fig. 5c show that after 1 day of co-cultivation, a noticeable amount of plaque covers both bare and KE coated tooth surfaces, while a smaller amount is observed on SAP coated surfaces. In contrast, SAP-KE coated tooth surfaces show scattered patches of plaque accumulation. Quantitative analyses illustrate that the average PPI of SAP-KE group (14 %) is significantly lower than that of bare group (72 %) after 1 day of co-cultivation (Fig. 5d). As co-culture time increases, the PPI of bare tooth surface remains at a high level, while those of SAP, KE, and SAP-KE groups gradually increase. Among these, SAP-KE group consistently maintains the lowest PPI. Throughout the 7-day observation period, the plaque on SAP-KE coated surface exhibits red staining at each time point, indicating the presence of fresh plaque, whereas the plaque on the other groups either remains or gradually turns blue staining, indicating the accumulation of old plaque. It is worth noting that after a single coating of SAP-KE and 7 days of co-culturing with bacteria, the tooth surface still maintains a slightly lower PPI compared to bare tooth surface co-cultured with bacteria for 1 day. These results demonstrate that even in the face of complex bacterial flora and simulated application environment, SAP-KE coating can offer sustained anti-fouling effect, remaining effective for up to 7 days under the simulated conditions. For special populations who are able to resume normal oral care within a week, such as patients who have undergone general oral surgery, a single application of SAP-KE peptide solution would be sufficient to effectively maintain oral hygiene.

Given that SAP-KE coating can be easily and quickly established on the tooth surface through spraying or rinsing, we further explore the maintenance effect of tooth surface cleaning after increasing its coating frequency. As shown in Fig. 5e, SAP-KE coated ex vivo teeth are co-cultured with dental plaque, removed every other day for coating supplementation, and stained with a plaque indicator for photo capture and PPI calculation on the 7th day. The results in Fig. 5f show that only a small amount of red-stained fresh plaque is present on the surface of SAP-KE coated tooth, while a large amount of dark blue-stained old plaque is found on bare tooth surface. The PPI analysis results show that, after supplementing SAP-KE coating every other day, the average PPI of coated tooth surfaces (24 %) is less than one third of that of bare tooth surfaces (82 %). These results suggest that increasing the frequency of SAP-KE coating application could improve the maintenance of tooth surface cleanliness. Therefore, for special populations who have difficulty maintaining normal oral care for more than a week, such as critically ill patients with low self-care ability or researchers in water-scarce environments, it is recommended to increase the application frequency of SAP-KE peptide solution to effectively reduce plaque adhesion and prevent the development of related diseases.

## 4 Conclusion

Inspired by dental plaque, we have developed a novel class of peptide SAP-KE that well integrates the advantage of SAP for rapid and selective adhesion to HA with that of KE for preventing protein and bacterial adhesion. Benefiting from its unique molecular structure and functional sequences, SAP-KE can quickly anchor to the tooth surface and form a robust anti-fouling coating, enabling efficient prevention of plaque accumulation without disrupting the balance of the oral microbiota. Our strategy provides a simple yet efficient new way for maintaining oral hygiene, particularly for populations who face challenges in performing regular mechanical tooth brushing. Hopefully, our work would provide an important direction for controlling plaque accumulation, as well as an application paradigm of the strategic wisdom of ‘A taste of your own medicine’ in scientific research.

## CRediT authorship contribution statement

**Huixue Wu:** Writing – original draft, Software, Project administration, Methodology, Formal analysis, Data curation. **Yiran Qin:** Methodology, Investigation, Formal analysis, Data curation. **Kexin Li:** Software, Methodology, Data curation. **Xinning Dai:** Visualization, Software. **Minghong Zhou:** Writing – review & editing. **Zongheng Cen:** Software, Methodology. **Yan Li:** Supervision, Funding acquisition, Conceptualization. **Zhike Huang:** Writing – review & editing, Supervision, Funding acquisition, Conceptualization. **Shuyi Wu:** Writing – review & editing, Writing – original draft, Project administration, Funding acquisition, Conceptualization.

## Declaration of competing interest

The authors declare that they have no known competing financial interests or personal relationships that could have appeared to influence the work reported in this paper.

## Data Availability

Data supporting the results of this study are available in the article and its supplementary information.
