# Supplementary material for: Dental plaque-inspired peptide engineered to control plaque accumulation
Source: Mater Today Bio. 2025 Feb 8;31:101570. doi: 10.1016/j.mtbio.2025.101570 (PMC11904560; doi:10.1016/j.mtbio.2025.101570)
Supplement: Multimedia component 1 [file mmc1.docx]

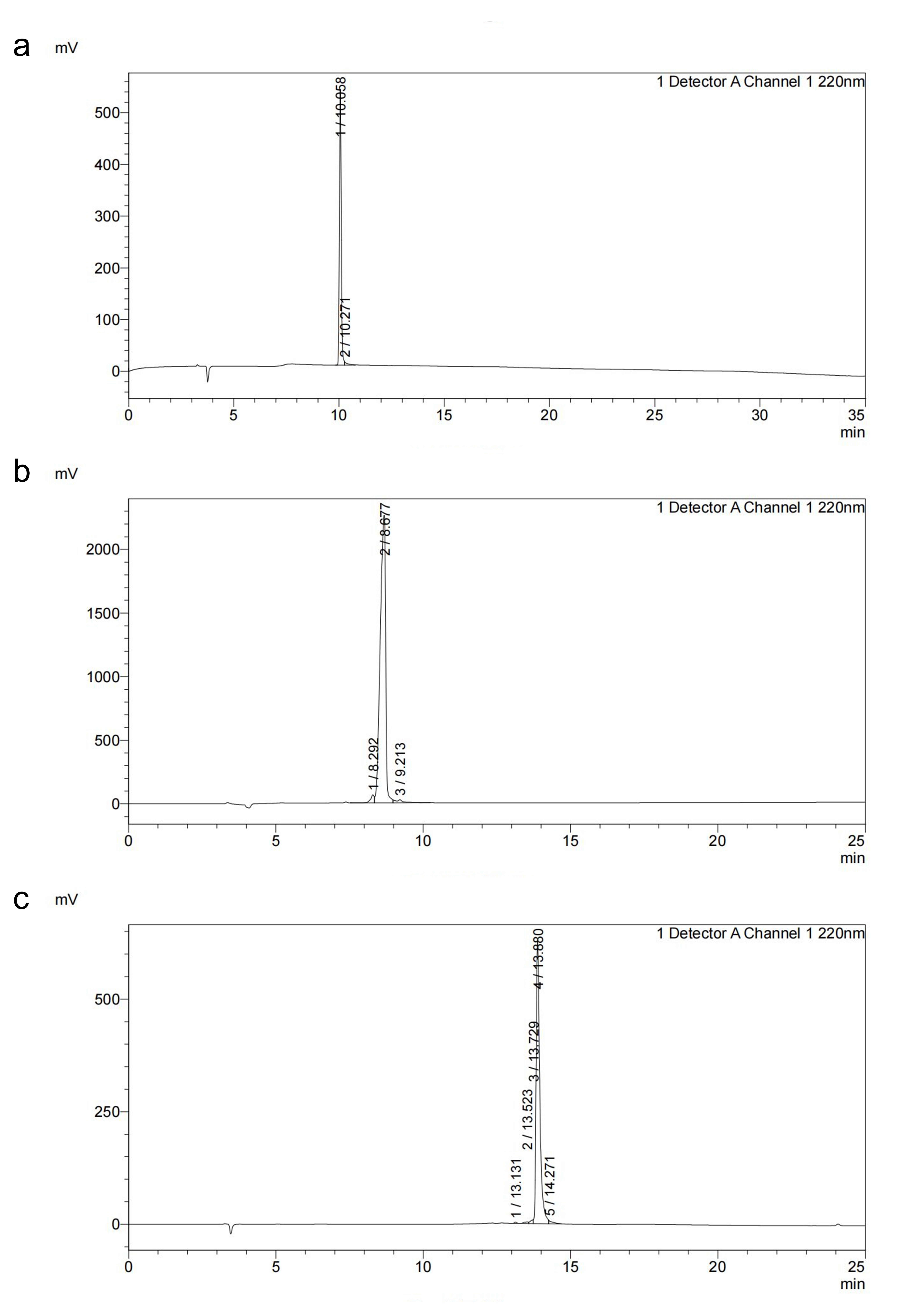


**Figure S1** HPLC chromatogram of KE (a), SAP (b), and SAP-KE (c) peptides.





**Figure S2** Mass spectra of KE (a), SAP (b), and SAP-KE (c) peptides.


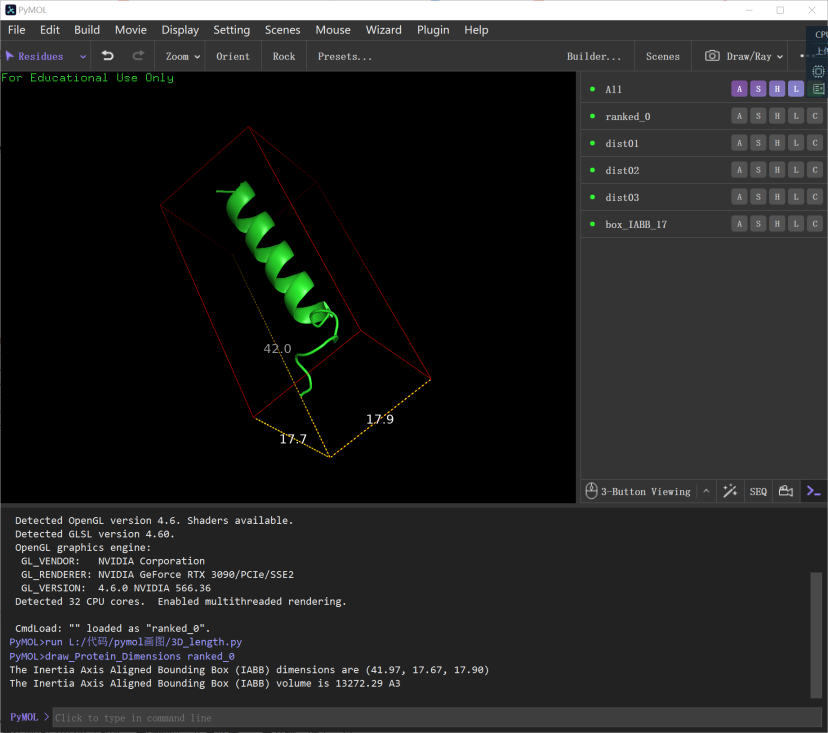

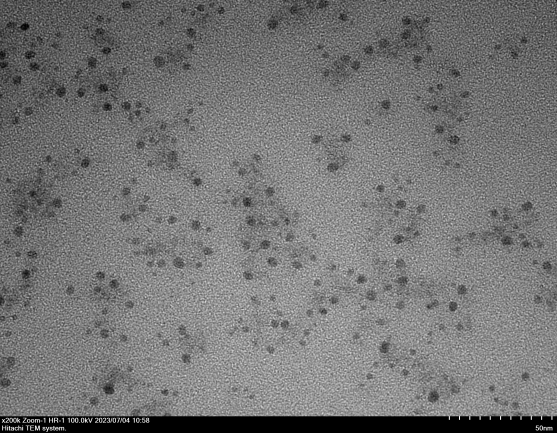


**Figure S3** (**a**) The three-dimensional image of SAP-KE peptide simulated by AlphaFold Protein Structure Database and PyMOL 2.4 software. (**b**) TEM image of SAP-KE peptides.


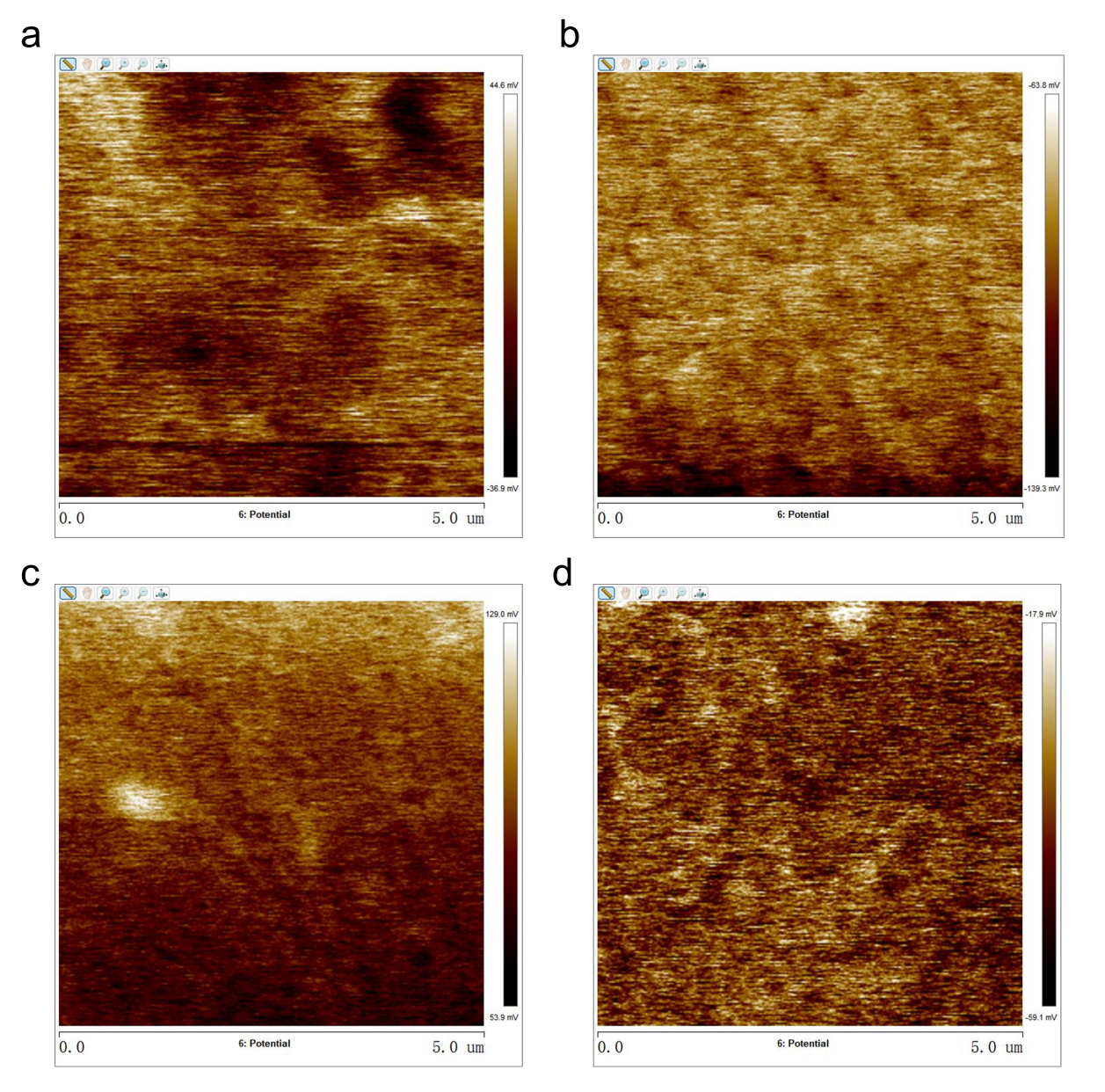


**Figure S4** KPFM images of bare (a), KE (b), SAP (c), and SAP-KE (d) coated HA.


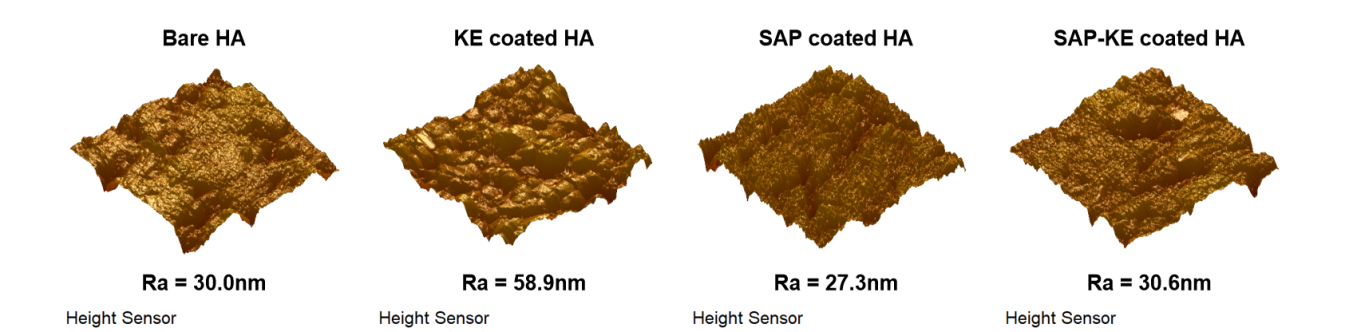


**Figure S5** AFM images of bare, KE, SAP, and SAP-KE coated HA.


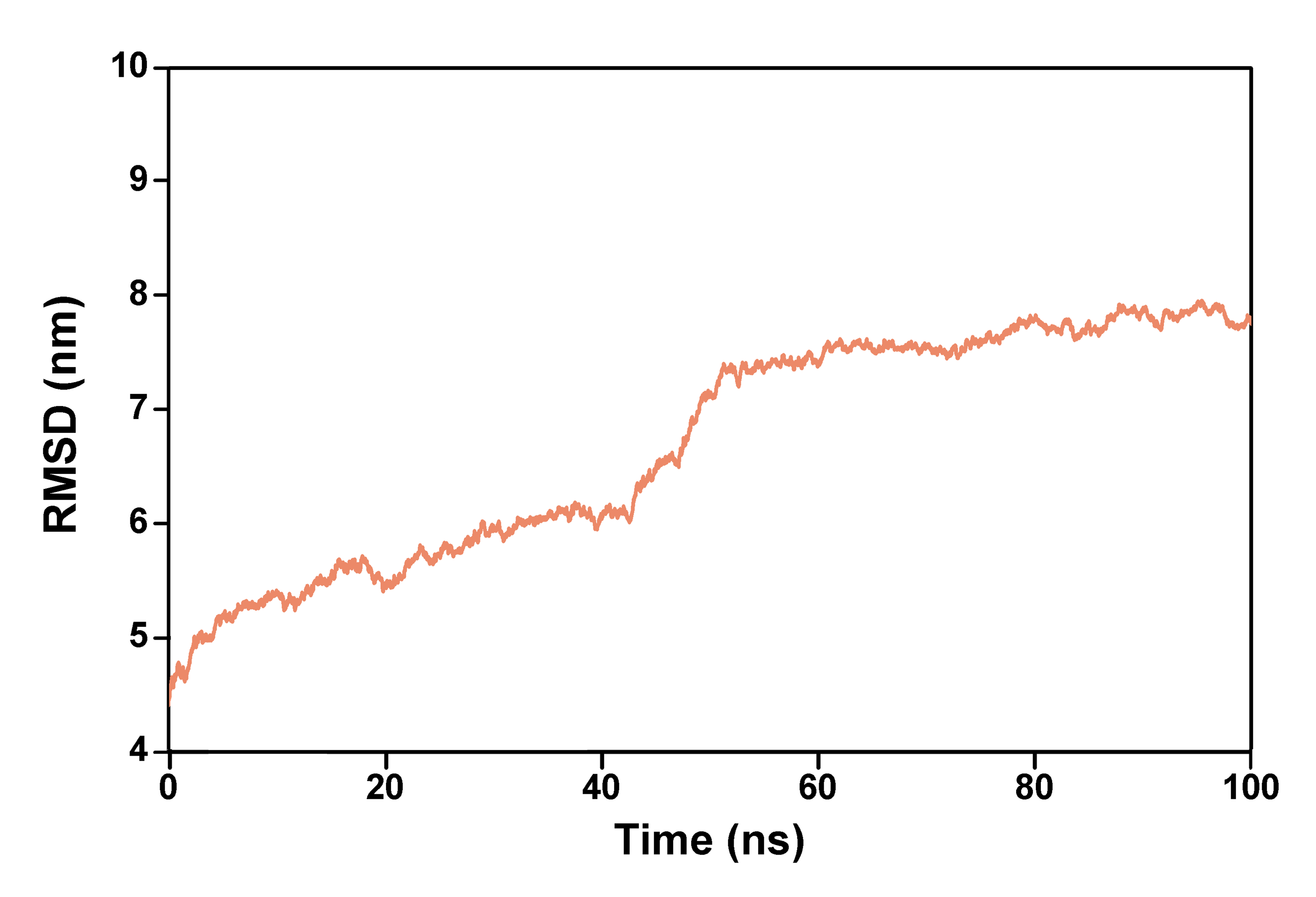


**Figure S6** Root mean square deviation (RMSD) of SAP-KE in the simulation system.
